# Clinical significance of epigenetic silencing and re-expression of O6-methylguanine-DNA methyltransferase using epigenetic agents in laryngeal carcinoma

**DOI:** 10.3892/ol.2014.2662

**Published:** 2014-11-03

**Authors:** JING YANG, XIN-BING ZHU, LI-XIA HE, ZHAO-WEI GU, MING-ZHU JIN, WEN-YUE JI

**Affiliations:** 1 Department of Otorhinolaryngology, Shengjing Hospital of China Medical University, Shenyang, Liaoning 110004, P.R. China; 2 Department of General Sugery, General Hospital of Liaohe Oil Field, Panjin, Liaoning 124010, P.R. China; 3 Department of Otorhinolaryngology, Fushun Second Hospital, Fushun, Liaoning 113001, P.R. China

**Keywords:** laryngeal carcinoma, O6-methylguanine-DNA methyltransferase gene, DNA methylation, histone modification, 5-aza-2′-deoxycytidine, trichostatin A

## Abstract

The aim of the present study was to investigate the association between O6-methylguanine-DNA methyltransferase (MGMT) gene expression levels, and DNA methylation status and histone modifications in laryngeal squamous cell carcinoma (LSCC). Chromatin immunoprecipitation, methylation-specific polymerase chain reaction (PCR), and reverse transcription-quantitative PCR were performed to analyze histone modifications, DNA methylation status and mRNA expression levels in the promoter region of the MGMT gene in laryngeal carcinoma HEp-2 cells, as well as in 50 paired healthy and LSCC tissue samples. The present study demonstrated that treatment of HEp-2 cells with 5-aza-2′-deoxycytidine (Aza), a DNA methyltransferase inhibitor, significantly upregulated MGMT mRNA expression levels, reduced MGMT DNA methylation, reduced MGMT histone H3 lysine 9 (H3K9) di-methylation, and increased MGMT histone H3 lysine 4 di-methylation without a significant change in H3K9 acetylation. Trichostatin A (TSA), a histone deacetylase inhibitor, marginally upregulated MGMT mRNA expression levels without affecting the DNA methylation status, or H3K9 or H3K4 di-methylation, however, TSA treatment caused a significant increase in H3K9 acetylation. Furthermore, Aza and TSA combination treatment produced a synergistic effect. In the LSCC samples, the rate of DNA methylation in the MGMT gene was 54%, compared with 24% in the healthy control group (P<0.05). Therefore, data from the present study indicates that MGMT may serve as a novel therapeutic target in the treatment of LSCC.

## Introduction

Laryngeal cancer is a common malignancy in otolaryngology, accounting for 1–5% of all cases of cancer, worldwide. It is the eleventh most common type of cancer, accounts for 35.4% of cases of head and neck cancer, and is the third most common type of head and neck malignancy, worldwide (1). O6-methylguanine-DNA methyltransferase (MGMT) is a key enzyme in the DNA repair network that removes mutagenic and cytotoxic adducts from O6-guanine in the DNA. Numerous carcinogens target O6-guanine, thus, the loss of MGMT gene expression results in the accumulation of unrepaired DNA damage and subsequent tumor development. MGMT is transcriptionally downregulated via the hypermethylation of CpG islands in its promoter region (2,3).

The average level of MGMT mRNA expression is significantly lower in cancerous mucosa compared with the corresponding non-cancerous mucosa. Histone modification is closely associated with the DNA methylation status of a gene and is key for gene regulation. DNA hypermethylation in the promoter CpG islands of tumor suppressor genes (TSGs) inhibits transcriptional initiation and results in permanent gene silencing, a key process in carcinogenesis (4–6). Histone H3 lysine 9 (H3K9) acetylation and histone H3 lysine 4 (H3K4) di-methylation are associated with active gene transcription, however, H3K9 di-methylation is associated with gene repression (7,8). Studies investigating the interaction between DNA methylation status and various histone modifications are currently ongoing.

To the best of our knowledge, no studies investigating the pattern of histone modifications in the TSG, MGMT in laryngeal carcinoma have been conducted. To establish a possible function for such epigenetic modifications of the MGMT gene in laryngeal carcinogenesis, the present report analyzed MGMT mRNA expression levels, DNA methylation status, and the levels of promoter region di-methyl-H3K9 (H3K9me2), H3K4me2 and acetyl-H3K9 (H3K9ac) following DNA methyltransferase inhibitor 5-aza-2′-deoxycytidine (Aza) and/or trichostatin A (TSA) treatment of laryngeal carcinoma HEp-2 cells. Furthermore, methylation-specific polymerase chain reaction (MSP) and reverse transcription (RT)-quantitative polymerase chain reaction (qPCR) were used to detect the association between MGMT gene expression levels and DNA methylation status in laryngeal squamous cell carcinoma (LSCC) tissues. Thus, the current report presents a mechanism for the inactivation of the TSG, MGMT in LSCC tissues.

## Materials and methods

### Cell line and tissue samples

HEp-2 cells were cultured in RPMI-1640 medium (pH 7.2; Gibco BRL, Life Technologies Inc., Grand Island, NY, USA) supplemented with 10% fetal bovine serum (inactivated under 56°C for 30 min), 100×10^3^ U/l penicillin and 100×10^3^ U/l streptomycin, and were cultured in a closed incubator in a 5% humidified CO_2_ atmosphere at a constant temperature of 37°C. Cells were required to reach the logarithmic growth phase and a viable cell count of 95–100% immediately prior to the experiments.

Fifty LSCC patients, who were diagnosed and treated between January 2008 and May 2009 at the Shengjing Hospital of China Medical University (Shenyang, China), were evaluated in the present study. Prior to surgery, the patients were pathologically diagnosed with LSCC; however, no chemotherapy or radiation was administered. Control mucosa samples were obtained from the patients who had received a total laryngectomy; the samples were obtained from tissue >2.0 cm from the tumor margin. Field cancerization may result in the tumor affecting a larger tissue area; therefore, only histologically healthy control mucosa samples were used in the present study. The samples were immediately snap-frozen in liquid nitrogen and stored at −80°C. Relevant clinical characteristics of the patients (age, gender, state of nodal metastases, and clinical stage, T stage and differentiation grade of the tumor) were extracted from the patients’ files. Tumor staging was conducted according to the International Union against Cancer 2002 tumor node metastasis classification (9). The patient tumor samples were collected following receipt of informed consent.

### Treatment of cells with Aza and TSA

HEp-2 cells were divided into three groups: (i) Aza group, 5 μmol/l Aza (Sigma-Aldrich, St. Louis, MO, USA) was added and cultured for 72 h; (ii) TSA group, 300 nmol/l TSA (Sigma-Aldrich) was added and cultured for 24 h; and (iii) Aza plus TSA group, 5 μmol/l Aza was added and cultured for 48 h prior to adding 300 nmol/l TSA and continuing to culture for 24 h. Control cells of the same batch, were not treated with any agent. The dose, time period and sequence of Aza and/or TSA treatment were based on similar preliminary studies (10–12).

### RNA extraction and RT-qPCR

Total RNA was isolated from cultured cells and tissues using TRIzol^®^ reagent (Invitrogen Life Technologies, Carlsbad, CA, USA) according to the manufacturer’s instructions. Total RNA treated with 2 μg DNase I (Thermo Fisher Scientific, Pittsburgh, PA, USA) was converted to complementary (c)DNA using a Reverse Transcription System kit (Thermo Fisher Scientific). RNA was excluded from the cDNA synthesis reactions and served as a negative control. PCR was performed in a 25-μl reaction volume using the Maxima SYBR^®^ Green/ROX qPCR Master Mix (Thermo Fisher Scientific) under the following conditions: 95°C for 10 min, followed by 40 cycles of 95°C for 30 sec (denaturation), 56°C for 30 sec (annealing), and 72°C for 30 sec (extension); and an additional extension of 72°C for 10 min. The PCR product length for the MGMT gene fragment was 151 bp and the MGMT primers were as follows: Upstream, 5′-CGAAATAAAGCTCCTGGGCA-3′ and downstream, 5′-GAACTCTTCGATAGCCTCGGG-3′. The PCR product length for the GAPDH gene fragment was 115 bp and the GAPDH primers were as follows: Upstream, 5′-TCCCATCACCATCTTCCAG-3′ and downstream, 5′-ATGAGTCCTTCCACGATACC-3′. The 2^−ΔΔct^ method was used to calculate the MGMT gene expression levels relative to an internal GAPDH control and, thus, the fold changes of the gene expression levels. All experiments were repeated three times for statistical analysis.

### MSP

Genomic DNA was prepared from cell lines and tissues using the phenol/chloroform extraction protocol and was modified by bisulfite treatment. Briefly, DNA was denatured by incubating with 0.3 mol/l NaOH at 37°C for 30 min, followed by incubation with 10 mmol/l hydroquinone and 3 mol/l sodium bisulfite (Sigma-Aldrich) at 55°C for 16 h. Modified DNA was purified using the Wizard DNA Clean-Up System (Promega Corporation, Madison, WI, USA) according to the manufacturer’s instructions. The MGMT PCR product length was 121 bp for the methylated fragment and 122 bp for the unmethylated fragment. The MSP primers are located in the promoter region of the MGMT gene and were as follows: Upstream, 5′-GGTCGTTTGTACGTTCGC-3′ and downstream, 5′-GACCGATACAAACCGAACG-3′ for the methylation primers; and upstream, 5′-GTAGGTTGTTTGTATGTTTGT-3′ and downstream, 5′-AACCAATACAAACCAAACA-3′ for the unmethylated primers. PCR was performed under the following conditions: 95°C for 12 min, followed by 32 cycles of 95°C for 30 sec (denaturation), 61°C for 45 sec (annealing) and 72°C for 30 sec (extension). An additional extension of 72°C for 7 min was performed prior to agarose gel electrophoresis and ethidium bromide staining. A Chemilmanger 5500 automatic image analyzer (Alpha Innotech Corporation, San Leandro, CA, USA) was used to obtain imaging data and analyze the electrophoresis results. All experiments were repeated three times for statistical analysis.

### Chromatin immunoprecipitation assay (ChIP)

ChIP was performed as previously described with specific modifications (13). Briefly, ~1.75×10^7^ HEp-2 cells, treated as described above, were fixed with 1% formaldehyde at 37°C for 20 min, resuspended in lysis buffer (1% sodium dodecyl sulfate, 10 mmol/l EDTA, 50 mmol/l Tris HCl; pH 8.1) and sonicated to generate ~500-bp DNA fragments. Antibodies against H3K9me2, H3K4me2 or H3K9ac (Upstate Biotechnology Inc., Lake Placid, NY, USA) were used to immunoprecipitate the major soluble chromatin fraction. The remaining soluble fraction was incubated with normal rabbit IgG (negative control) and used as a DNA input control. DNA-protein crosslinks were reversed by heating the samples to 65°C for 5 h and digesting with proteinase K. DNA was then extracted using the phenol/chloroform protocol. The ChIP experiments were repeated three times.

### PCR analysis of the immunoprecipitated DNA

PCR reactions were performed using 2 μl immunoprecipitated DNA, a negative control and a DNA input control. The PCR product length for the MGMT gene fragment was 171 bp and the ChIP-PCR primers, located in the MGMT promoter region, were as follows: Upstream, 5′-CCCCATCTCCAAATAAGGTCA-3′ and downstream, 5′-CCTAGACACTGCCAGAGCCTG-3′. PCR products were resolved on 2% agarose gels (Promega Corporation) and quantified using a GelDoc 1000 (Bio-Rad, Hercules, CA, USA) and Molecular Analyst software (Alpha Innotech Corporation). The levels of H3K9, and H3K4 di-methylation and H3K9 acetylation in each immunoprecipitate were determined by quantifying the intensities of the PCR products in the immunoprecipitated versus input DNA. ChIP was repeated a minimum of two times and three independent PCR analyses of each sample were performed.

### Wound healing assay

HEp-2 cells were seeded in six-well plates and cultured in RPMI-1640 medium supplemented with 10% fetal bovine serum. A wound was created in the center of the cell monolayer using a sterile plastic pipette tip. The cells were cultured for 24 h to allow cell migration. To assess the ability of the cells to migrate into the wound area, an inverted microscope was used to capture images of the cells 0 and 24 h after wounding.

### Matrigel^®^ invasion assay

HEp-2 cells (5×10^4^) cultured in 200 μl serum-free RPMI-1640 medium were seeded onto the upper chambers of Matrigel^®^-coated Transwell^®^ filters (pore size, 8 μm; Corning Life Sciences, Corning, NY, USA). RPMI-1640 (500 μl) supplemented with 10% fetal bovine serum was added to the lower chambers as a chemoattractant. Cells were incubated at 37°C in a humidified 5% CO_2_ atmosphere for 24 h. Cells that had successfully invaded through the inserts were fixed in 4% paraformaldehyde for 30 min and stained with methylrosanilinium chloride. The invaded cells were counted from five preselected microscopic fields (magnification, ×200). The mean result of the assay was obtained from three independent experiments.

### Statistical analysis

The ratio results were expressed as the mean ± standard deviation. Student’s t-test was used to calculate the significance between the treated and control samples. The Spearman rank-correlation test was used to examine the association between the MGMT expression level, and DNA hypermethylation and histone modification. Furthermore, χ^2^ and Fisher’s exact tests were adopted to analyze the aberrant DNA hypermethylation of MGMT within the clinicopathological parameters. Statistical calculations were performed using SPSS version 13.0 (SPSS Inc., Chicago, IL, USA) and P<0.05 was considered to indicate a statistically significant difference.

## Results

### Analysis of MGMT mRNA expression levels and DNA methylation status in laryngeal carcinoma HEp-2 cells before and after treatment with Aza and TSA

**Figure 1:**
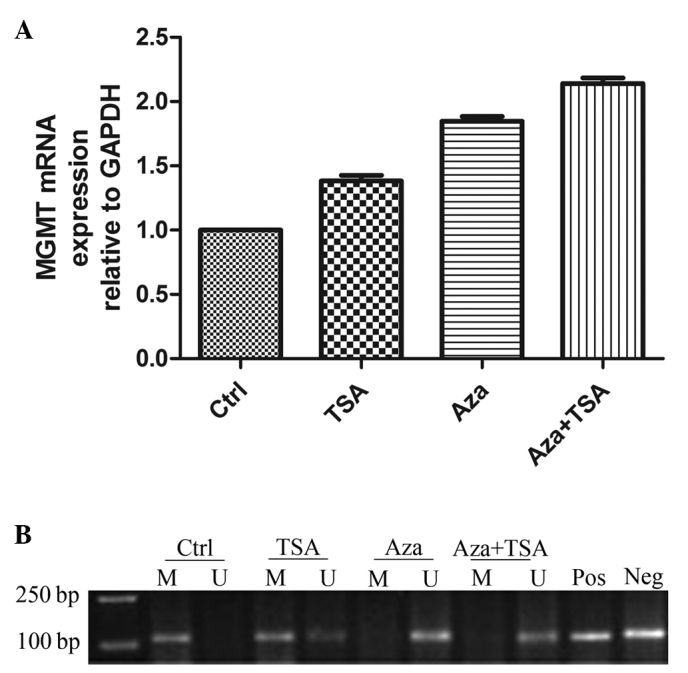
MGMT mRNA expression levels and DNA methylation status in laryngeal carcinoma HEp-2 cells before and after treatments with Aza and TSA. (A) RT-qPCR analysis of MGMT mRNA expression in laryngeal carcinoma HEp-2 before (Ctrl) and after treatment with Aza and/or TSA. (B) Methylation-specific PCR analysis of DNA methylation at the MGMT promoter region in laryngeal carcinoma HEp-2 cells before and after treatment with Aza and/or TSA. Lane M, methylated alleles (121 bp); lane U, unmethylated alleles (122 bp); Pos, positive control; Neg, negative control. A minimum of three independent experiments were performed, which all produced similar results. MGMT, O6-methylguanine-DNA methyltransferase; Ctrl, control; TSA, trichostatin A; Aza, 5-aza-2′-deoxycytidine; RT-qPCR, reverse transcription-quantitative polymerase chain reaction.

RT-qPCR was performed to assess the level of MGMT mRNA expression in the control group (no Aza or TSA treatment; relative mRNA expression level, 1) compared with the expression level following treatment with TSA (relative mRNA expression level, 1.383±0.0417; P<0.05), Aza (relative mRNA expression level, 1.847±0.0.03844; P<0.01) and a combination of Aza and TSA (relative mRNA expression level, 2.140±0.04509; P<0.01; Fig. 1A).

To identify whether DNA methylation was associated with the changes in MGMT mRNA expression level, the present study used MSP to assess the DNA methylation status of MGMT in HEp-2 cells. DNA methylation in the MGMT gene promoter region (presence of a methylation band only) was apparent prior to treatment. TSA treatment induced hemi-methylation of the MGMT promoter CpG islands (co-presence of methylation and non-methylation bands) and Aza treatment resulted in total DNA demethylation (presence of a methylation band only). The effect of Aza and TSA combination treatment was similar to that of Aza alone (Fig. 1B).

### Differential effects of Aza and TSA treatment on histone modification in laryngeal carcinoma HEp-2 cells

**Figure 2:**
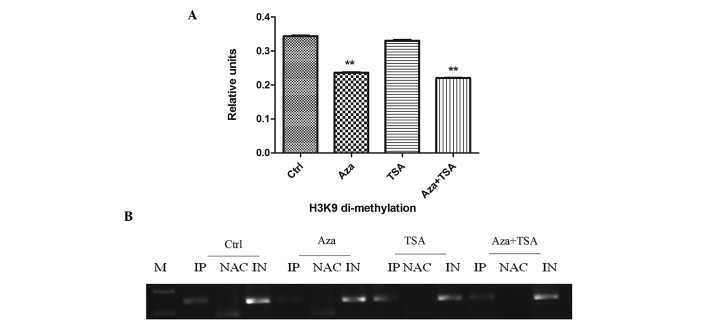
ChIP analysis of H3K9 di-methylation before and after treatment of HEp-2 cells with Aza, TSA, or Aza and TSA. Three independent ChIP assays were performed using an antibody that recognizes di-methyl H3K9 at the O6-methylguanine-DNA methyltransferase promoter region. (A) Summary of PCR analyses of ChIP assays. The mean precipitated DNA/input DNA ratios demonstrated on the y-axis represent the relative values of H3K9 di-methylation. Mean H3K9 di-methylation levels are demonstrated by the standard error bars and ^**^P<0.01. (B) PCR assay. Ctrl, prior to treatment; Aza, following treatment with Aza; TSA, following treatment with TSA; Asa + TSA, following treatment with Aza and TSA. ChIP, chromatin immunoprecipitation; H3K9, histone 3 lysine 9; Aza, 5-aza-2′-deoxycytidine; TSA, trichostatin A; PCR, polymerase chain reaction; IP, immunoprecipitated DNA; NAC, no antibody control; IN, input DNA from whole cell lysate.

**Figure 3:**
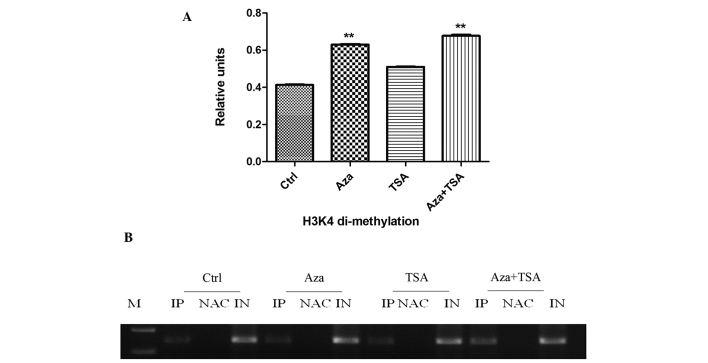
ChIP analysis of H3K4 di-methylation before and after treatment of HEp-2 cells with Aza, TSA, or Aza and TSA. Three independent ChIP assays were performed using an antibody that recognizes di-methyl H3K4 at the O6-methylguanine-DNA methyltransferase promoter region. (A) Summary of PCR analyses of ChIP assays. The mean precipitated DNA/input DNA ratios demonstrated on the y-axis represent the relative values of H3K4 di-methylation. Mean H3K4 di-methylation levels are demonstrated by the standard error bars and ^**^P<0.01 vs. control group. (B) PCR assay. Ctrl, prior to treatment; Asa, following treatment with Aza; TSA, following treatment with TSA; Asa + TSA, following treatment with Aza and TSA. ChIP, chromatin immunoprecipitation; H3K4, histone 3 lysine 4; Aza, 5-aza-2′-deoxycytidine; TSA, trichostatin A; PCR, polymerase chain reaction; IP, immunoprecipitated DNA; NAC, no antibody control; IN, input DNA from whole cell lysate.

**Figure 4:**
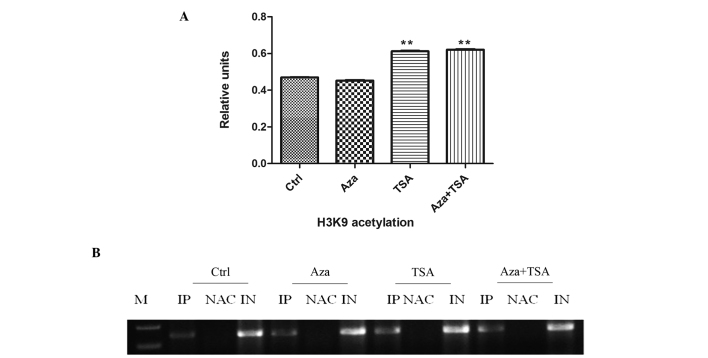
ChIP analysis of H3K9 acetylation before and after treatment of HEp-2 cells with Aza, TSA, or Aza and TSA. Three independent ChIP assays were performed using an antibody that recognizes H3K9 acetylation at the O6-methylguanine-DNA methyltransferase promoter region. (A) Summary of PCR analyses of ChIP assays. The mean precipitated DNA/inputDNA ratios demonstrated on the y-axis represent the relative values of H3K9 acetylation. Mean H3K9 acetylation levels are demonstrated by the standard error bars and ^**^P<0.05 vs. control group. (B) PCR assay. Ctrl, prior to treatment; Asa following treatment with Aza; TSA, following treatment with TSA; Asa + TSA, following treatment with Aza and TSA. ChIP, chromatin immunoprecipitation; H3K9, histone 3 lysine 9; Aza, 5-aza-2′-deoxycytidine; TSA, trichostatin A; PCR, polymerase chain reaction; IP, immunoprecipitated DNA; NAC, no antibody control; IN, input DNA from whole cell lysate..

To elucidate the effect of epigenetic agents on histone modifications, HEp-2 cells were treated with Aza and TSA. Aza treatment resulted in a significant decrease in the levels of H3K9 di-methylation in the MGMT promoter region (0.343±0.003 vs. 0.236±0.001; P<0.01) and TSA treatment resulted in a marginal reduction in H3K9 di-methylation (0.343±0.003 vs. 0.330±0.003; P>0.05). The effect of combined Aza and TSA treatment on H3K9 di-methylation was similar to that of Aza alone (0.343±0.003 vs. 0.220±0.001; P<0.01; Fig. 2). To analyze the level of H3K4 di-methylation at MGMT promoter regions, ChIP assays were performed on HEp-2 cells. Aza treatment significantly increased the level of H3K4 di-methylation at the MGMT promoter (0.484±0.007 vs. 0.631±0.002; P<0.01); however, TSA treatment did not affect H3K4 di-methylation (0.484±0.007 vs. 0.510±0.002; P>0.05; Fig. 3). The effect of combined Aza and TSA treatment on the level of H3K4 di-methylation was similar to that of Aza alone (0.484±0.007 vs. 0.677±0.006; P<0.01). Furthermore, H3K9 acetylation was significantly increased at MGMT promoter regions following treatment with TSA (0.470±0.001 vs. 0.612±0.003; P<0.01), however, Aza exhibited no marked effect on H3K9 acetylation (0.470±0.001 vs. 0.452±0.002, P>0.05). The effect of combined Aza and TSA treatment on H3K9 acetylation levels was similar to that of TSA alone (0.470±0.0007 vs. 0.621±0.0023, P<0.05; Fig. 4).

### Frequent DNA methylation of MGMT in LSCC and its clinical significance

**Figure 5:**
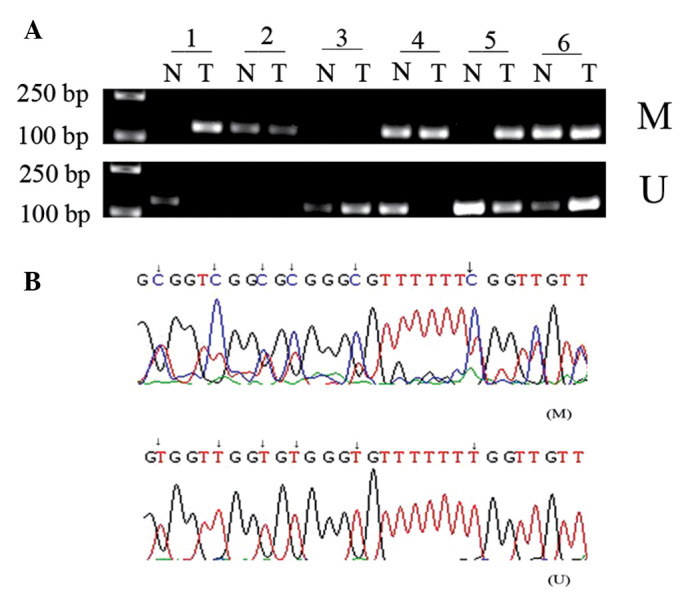
Methylation status of the MGMT gene promoter in laryngeal carcinoma tissues and the paired adjacent healthy tissues. (A) Methylation-specific polymerase chain reaction demonstrates the DNA methylation pattern of MGMT in laryngeal squamous cell carcinoma and the corresponding healthy tissue samples. 1–6, case sample number; lane N, non-malignant laryngeal tissue; lane T, laryngeal carcinoma tissues; M, methylation bands; U, unmethylated bands. (B) Demonstration of MGMT promoter methylation by sequencing following sodium bisulfite modification. MGMT, O6-methylguanine-DNA methyltransferase.

The methylation status of MGMT in LSCC tissue and paired adjacent healthy tissue samples was detected using MSP. The DNA methylation rate of MGMT was significantly higher in laryngeal cancer (54%) compared with 24% in the healthy control group (P<0.05; Fig. 5A; data not shown). The methylated and unmethylated products of MSP were sequenced, confirming that sodium bisulfite modification was sufficient for the DNA (Fig. 5B). The present study also identified that the DNA methylation status of MGMT exhibited no correlation with the age or gender of the patient, the degree of tumor differentiation, the tumor T stage or lymph node metastasis in LSCC tissues (P>0.05; Table I).

### Low expression level of MGMT is associated with DNA methylation status in LSCC tissues

**Figure 6:**
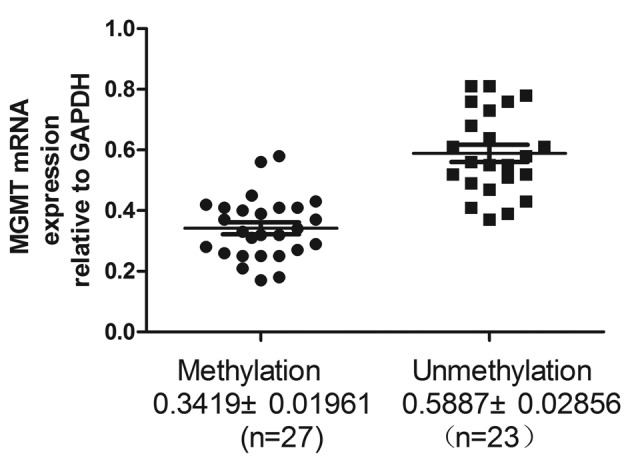
Reverse transcription-quantitative polymerase chain reaction analysis of MGMT mRNA expression levels in MGMT methylated and unmethulated laryngeal squamous cell carcinoma tissue. MGMT, O6-methylguanine-DNA methyltransferase.

RT-qPCR was performed to assess the MGMT mRNA expression level in LSCC tissues. The present study identified that MGMT mRNA expression levels were significantly lower in LSCC tissues exhibiting MGMT DNA methylation compared with unmethylated DNA (0.3419±0.01916 vs. 0.5887±0.02856; P<0.0001; Fig. 6 and Table II).

### Migration and invasion inhibition of HEp-2 cells following treatment with Aza

**Figure 7:**
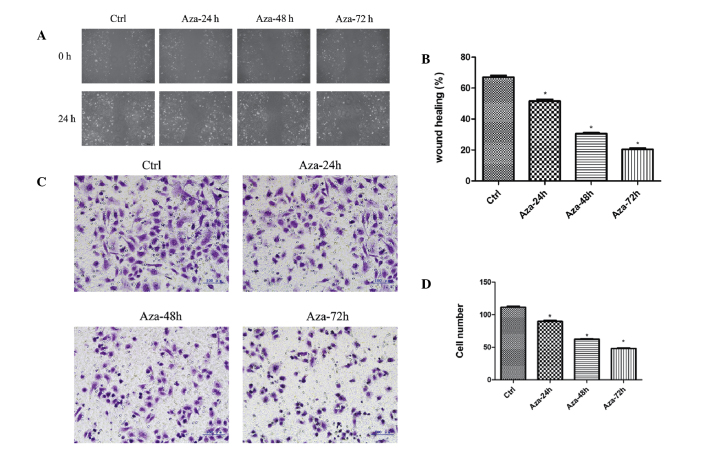
Effect of Aza on the migration and invasion of HEp-2 cells *in vitro*. (A) Wound healing assay of HEp-2 cells. Images were captured 24 h after wounding (magnification, ×100). (B) Percentage wound closure was measured in at least three randomly selected regions (means ± SD). (C) Invasion ability of HEp-2 cells was determined by a Matrigel^®^ invasion assay following treatment with Aza. Representative images of treated and untreated cells are presented (magnification, ×200). (D) Number of cells invading the wounded area at 24 h (means ± SD). ^*^P<0.01 vs. Ctrl. Aza, 5-aza-2′-deoxycytidine; Ctrl, control; SD, standard deviation.

To investigate the inhibitory effect of Aza on the migration and invasion of HEp-2 cells, a wound-healing and Matrigel^®^ invasion assay was performed. The present study demonstrated that 24 h after establishing the wound, the control group achieved the most wound closure (67.00±1.080%), compared with the Aza 24- (51.50±1.190%), 48- (30.50±0.6455%) and 72-h (20.50±0.6455%) groups (F=510.9; P<0.0001; Fig. 7A and B). The Matrigel^®^ invasion assay demonstrated that the number of invading HEp-2 cells was 111.5±1.190, 89.75±1.250, 62.25±0.4787, and 48.00±0.9129% in the control, Aza 24-, 48- and 72-h groups, respectively. The number of invading HEp-2 cells was significantly reduced following Aza treatment when compared with the control group (F=794.5; P<0.0001; Fig. 7C and D). Thus, Aza significantly reduced HEp-2 cell migration and invasion.

## Discussion

A growing number of TSGs are being identified that are inactivated by epigenetic, rather than classic, mutation/deletion events (14,15). Unlike mutational inactivation, methylation is reversible, thus, demethylating agents and inhibitors of histone deacetylases (HDACs) have been evaluated in clinical trials (16–19). Aza, a pyrimidine analogue with the 2′-deoxycytidine fifth carbon atom replaced by a nitrogen atom, binds to DNA molecules during replication and subsequently forms a complex with DNA methyltransferase (DNMT1). This complex inhibits the transmethylation activity of DNMT1. TSA is a HDAC inhibitor that causes DNA histone hyperacetylation and subsequently induces p21 (WAF1/CIP1) gene expression. Upregulation of p21 (WAF1/CIP1) gene transcription may result in cell cycle arrest and inhibition of cell growth by regulating cell cycle regulatory factors and the expression levels of apoptosis-associated proteins (17,20).

In the present study, it was identified that DNA hypermethylation-silenced MGMT in laryngeal carcinoma HEp-2 cells is characterized by H3K9 hypermethylation, and H3K9 and H3K4 hypomethylation at the promoter. Following treatment with Aza alone or in combination with TSA, H3K9 di-methylation decreased and H3K4 di-methylation increased at the promoter, consistent with DNA demethylation and reactivation of MGMT expression levels. In addition, the effect of combined Aza and TSA treatment on MGMT expression levels was stronger than that of Aza treatment alone. Treatment of laryngeal carcinoma HEp-2 cells with Aza appeared to reverse the MGMT gene methylation status and demethylate the promoter region. Furthermore, the effect of combined Aza and TSA treatment on MGMT expression levels was stronger than that of Aza treatment alone. In the present study, the MGMT methylation status was not affected by TSA alone as TSA is an HDAC inhibitor, which does not affect the gene promoter methylation status. The primary effect of epigenetic agents on MGMT gene expression levels was promoter hypermethylation: MGMT gene expression level was significantly upregulated by Aza, marginally upregulated by TSA, and synergistically upregulated using a combination of the two epigenetic agents. The results indicate that the primary epigenetic factor influencing gene expression levels and downregulating the TSG transcription was MGMT gene DNA methylation, however, histone modifications were also key. ChIP assays were conducted to investigate the association between the DNA methylation status and histone modifications. ChIP is a technique used to identify the presence of particular DNA-binding proteins that may modulate chromatin structure and/or transcriptional characteristics of the specific region of DNA with which the DNA-binding proteins are associated. The present study demonstrated that H3K9 di-methylation levels in the MGMT promoter region correlated with the DNA methylation status. MGMT reactivation by Aza was accompanied by DNA demethylation and a decrease in H3K9 di-methylation levels. In contrast to H3K9 di-methylation, H3K4 di-methylation in the promoter region was inversely correlated with the DNA methylation status. Furthermore, H3K4 di-methylation may be associated with an open chromatin configuration and transcriptional activation (21). Aza increased the level of H3K4 di-methylation, however, there was no significant affect on H3K9 acetylation. TSA significantly increased the level of H3K9 acetylation in HEp-2 cells, although it produced a marginal effect on MGMT gene expression levels. The present study proposes that DNA methylation may be important in gene silencing and the maintenance of repressive histone modifications at hypermethylated gene promoters in laryngeal carcinogenesis. It has previously been reported that DNA modification itself, or components of the DNA methylation machinery, such as DNMTs or methyl CpG-binding proteins, may directly interact with histone methyltransferases or proteins in regions of DNA methylation. This interaction allows the DNA methylation machinery to assemble an alterative histone modification, demonstrating that histone methylation depends on DNA methylation (22,23).

The DNA methylation status of a gene has previously been reported as a promising biomarker in the early diagnosis and prognosis of cancer (24,25). Aberrant DNA hypermethylation of gene promoter regions is an important epigenetic mechanism that regulates gene expression levels, resulting in the downregulation and silencing of various TSGs (26–28). In the present report, the MGMT gene was identified to exhibit a frequent methylation rate in LSCC, which may indicate that the occurrence of laryngeal cancer is associated with promoter methylation of the MGMT gene. The MGMT mRNA expression level is significantly lower when the promoter is methylated compared with when the promoter is unmethylated. Therefore, the mRNA expression level of the TSG, MGMT exhibits a negative correlation with CpG island methylation in LSCC. Furthermore, the MGMT methylation status exhibits no correlation with patient age or gender, tumor differentiation degree, tumor T stage or lymph node metastasis in LSCC. This lack of correlation indicates that MGMT gene methylation is not limited to a particular stage or sub-type of LSCC, but is involved in the entire development process. In addition, the present study examined the effect of Aza application on the migration and invasion ability of HEp-2 cells using wound-healing and Matrigel^®^ invasion assays. Aza application inhibited the migration and invasion ability of HEp-2 cells, indicating that DNA demethylation may inhibit the invasion ability of HEp-2 cells. Recently, Aza was demonstrated to synergize with progesterone therapy to inhibit endometrial cancer cell growth and invasion (29). Thus, additional studies are required to fully elucidate the potential of epigenetic agents in cancer therapy.

In conclusion, the present study demonstrates that frequent epigenetic alterations regulate MGMT gene expression level in LSCC. The data provides a foundation for further investigations into the role of the MGMT gene in laryngeal carcinoma and its potential as a biomarker in the early diagnosis, treatment and prognosis of laryngeal carcinoma.

## Figures and Tables

**Table I:** Clinicopathological parameters and MGMT methylation status of tissue samples from laryngeal squamous cell carcinoma patients.

Variable	Patients, n	MGMT methylation status, n (%)		P-value

		M	U	
Gender				0.517
Male	44	23 (52.3)	21 (47.7)	
Female	6	4 (66.7)	2 (33.3)	
Age, years				0.162
<60	24	16 (66.7)	8 (33.3)	
≥60	26	11 (42.3)	15 (57.7)	
Tumor T stage				0.982
T1 + T2	24	13 (54.2)	11 (45.8)	
T3 + T4	26	14 (53.8)	12 (46.2)	
Lymphatic metastasis				0.968
Positive	11	6 (54.5)	5 (45.5)	
Negative	39	21 (53.8)	18 (46.2)	
Tumor Grade				0.586
Well-differentiated	29	18 (62.0)	11(38.0)	
Moderately and poorly differentiated	21	9 (42.9)	12 (57.1)	
Type				0.747
Glottic	23	13 (56.5)	10 (43.5)	
Supraglottic	27	14 (51.9)	13 (47.1)	

P<0.05 was considered to indicate a statistically significant difference. The χ^2^ test was used to analyze the data. MGMT, O6-methylguanine-DNA methyltransferase; M, methylated and partially methylated; U, unmethylated.

**Table II:** Association between MGMT mRNA expression levels and DNA methylation status in laryngeal squamous cell carcinoma tissues.

DNA methylation status	Cases, n	MGMT mRNA expression level
Methylated	27	0.3419±0.01961
Unmethylated	23	0.5887±0.02856

The student’s t-test was used to calculate the significance of differences in MGMT mRNA expression level between the methylated and unmethylated groups (t=17.628; P<0.0001) MGMT, O6-methylguanine-DNA methyltransferase.
